# Improving the assessment of older adult’s nutrition in primary care: recommendations for a proactive, patient-centred and aetiology approach

**DOI:** 10.1136/bmjnph-2023-000661

**Published:** 2023-11-02

**Authors:** Rebecca Fisher, Kathy Martyn, Vittoria Romano, Alison Smith, Rosemary Stennett, Sally Ayyad, Sumantra Ray

**Affiliations:** 1 NHS London Procurement Partnership, Guy's and St Thomas' NHS Foundation Trust, London, UK; 2 School of Sport and Health Science, University of Brighton, Brighton, UK; 3 NNEdPro Global Centre for Nutrition and Health, St John’s Innovation Centre, Cambridge, UK; 4 Central London Community Healthcare NHS Trust, London, UK; 5 Hertfordshire and West Essex Integrated Care Board, Hertfordshire, UK; 6 North Central London Integrated Care System, London, UK; 7 School of Biomedical Sciences, Ulster University, Coleraine, UK; 8 Fitzwilliam College, University of Cambridge, Cambridge, UK

**Keywords:** malnutrition, preventive counselling, musculo-skeletal health, nutrition assessment

## Background

### The nature of the problem

**Figure 1:**
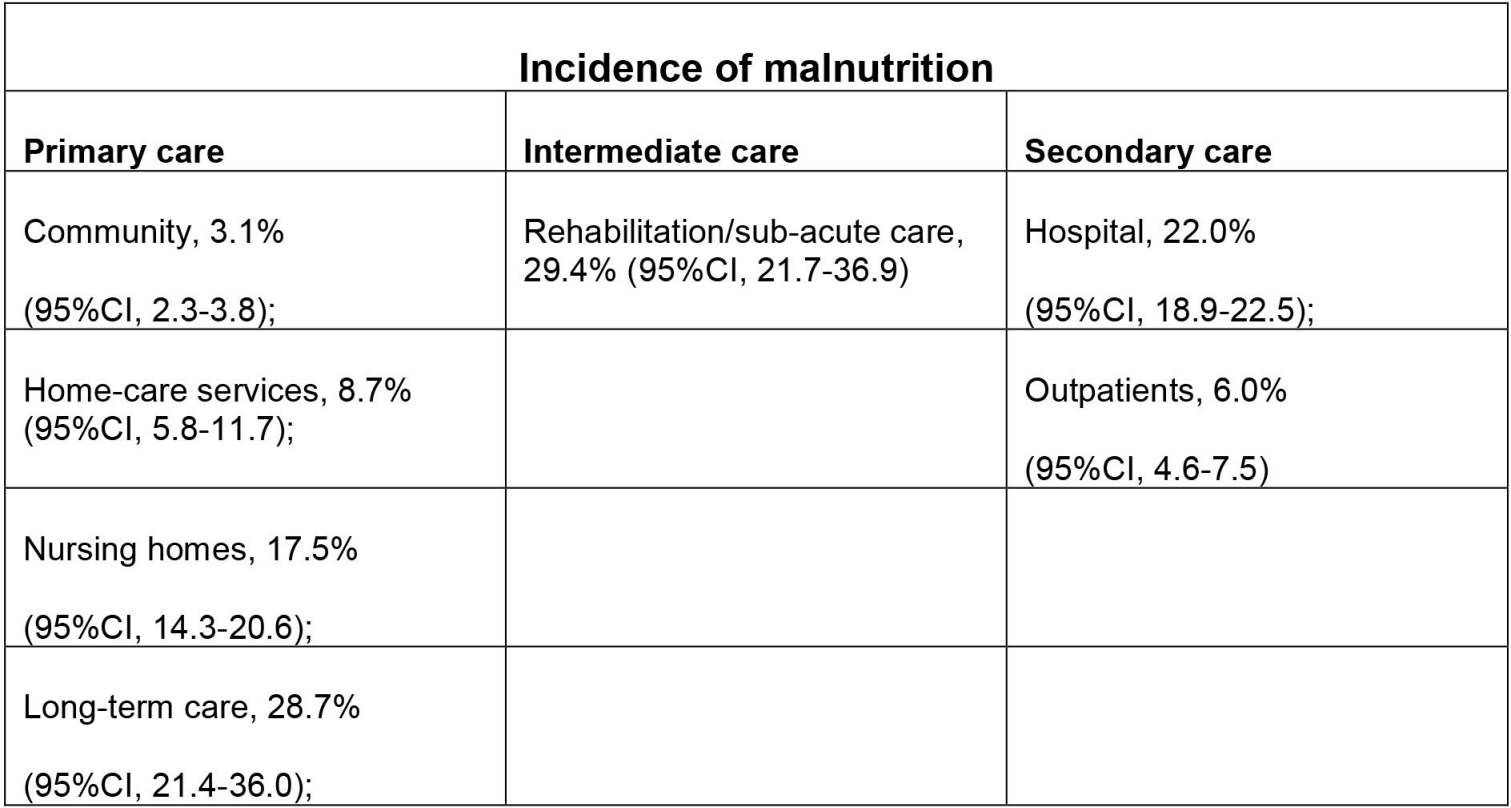
Incidence of malnutrition by setting, adapted from Cereda *et al.* 37

The UK has an ageing population, with the greatest increase in those aged over 85.^1^ While most will have long periods of good health, many will live with long-term conditions, and some will become frail requiring continued support in the community or in care and nursing homes.^2^ Prevalence of malnutrition in older adults differs significantly, depending on the healthcare setting (figure 1).

Causes of malnutrition in older people are multifactorial,^3^ some such as vitamin B_12_ deficiency are age related, and others such as low vitamin D status reflect reduced mobility impacting time spent outdoors.^4^ Also, older people are more likely to be prescribed multiple medications^5^ may be underhydrated, suffer alterations to taste and swallowing, have reduced access to food, or difficulties in shopping for, and preparing meals. In conditions, such as chronic obstructive pulmonary disease, inflammation-induced catabolism is greater, with reduced weight and increased risk of sarcopenia is prevalent.^6^ Patients may also present with metabolic syndrome issues or obesity with sarcopenia.^7^

There is overlap between malnutrition, sarcopenia, frailty^8^ and functional ability.^9^ A systematic review of 178 644 participants shows that unintentional weight loss has a significant impact on mortality regardless of overweight or obesity, and risk is greater with age.^10^

We recognise the complexities of the health economics evidence base including the implications of funding and publication bias, the relative lack of studies in primary care and challenges of demonstrating outcomes for a diverse array of non-commercial interventions. The heterogeneity in the evidence base for nutritional interventions is challenging. The Nutrition Education Policy for Health Care Practice initiative of the NNEdPro Global Institute seeks to disseminate evidence informed practice patterns to build more precise nutrition capacity within the healthcare workforce. We propose this biopsychosocial model practice pattern as a pragmatic and ethical approach to improve the nutrition of older adults by earlier identification of malnutrition and initiating relevant actions by any healthcare professional (HCP).

### Limitations of current approaches

Currently, there is a gap between the recommendations to screen and treat for malnutrition^11 12^ and shared understanding of the problem of unintentional weight loss and frailty by older adults,^13–15^ carers^15^ and HCPs.^16–18^ There is a lack of implementation and limited evidence base for the impact of screening and management on outcomes in different clinical populations including primary care.^19 20^ The Canadian Malnutrition Task Force–Primary Care Working Group reviewed literature and best practice to develop consensus interdisciplinary care for adults in the community.^21^ We seek to approach the issue differently by proposing the use of an existing tool developed by and for the community that addresses patient concerns.

There are many nutritional dilemmas, such as effective management of frailty and unintentional weight loss, whether and when to relax historical dietary advice, defining healthier eating for older adults and appropriateness of prescription of Oral Nutritional Supplements (ONS). A further dilemma is the lack of agreed measures for frailty and malnutrition.^22^ The role of biochemical tests is of limited value unless there is clinical suspicion of a specific micronutrient deficiency or the need to exclude sinister causes of weight loss such as cancer.^23^ Recommendations on specific dietary interventions are challenging due to limitations in the evidence base.^8 24 25^ Many existing pathways follow a biomedical approach to managing undernutrition by providing additional nutrients (via food-based management, prescribed ONS or a combination of the two) without a holistic assessment of basic underlying issues. The Cochrane review^24^ observed that benefits might be due to improved levels of care and multiple randomised control trials comparing dietary advice with ONS found little or no difference in weight (<6 months), with one study suggesting advice may lead to better quality of life (12 months).^24^ Patient representatives questioned the significance of the measures investigated and clinical outcomes, in comparison to what matters to them.^26^

**Figure 2:**
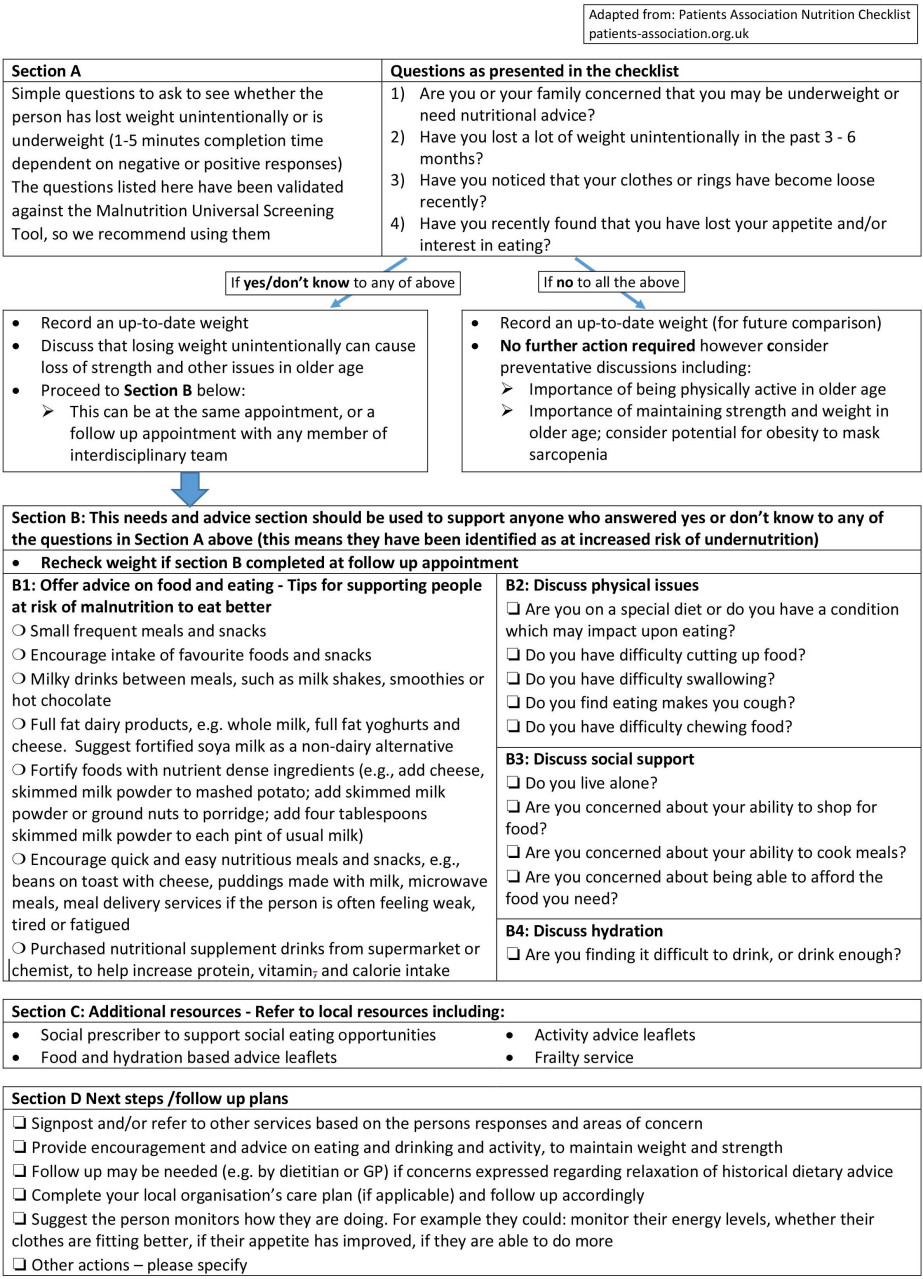
Patient association nutrition checklist. The screening questions (section A) have been validated against the Malnutrition Universal Screening Tool (MUST).^30^ The checklist (section B) is holistic and reflects areas of concern that are taught in dietetic curricula, and are relevantfor all health professionals. The development process is fully described, involved patients and carers in the UK, and has undergone evaluation and refinement to improve).^29–31^ We note that some industry funding has contributed towards the development of the checklist. However, the robust process, led by Wessex Academic Health Science Network addresses a lack of researched, peer reviewed and evaluated practices in this area. Evaluation of the practice is required, beyond user feedback, to determine how implementation affects a range of outcomes. Additional questions/prompts have been included. Firstly, derived from the United States Department of Agriculture household food security tool validated for use in clinical settings to address our concern with the intersectionality of food insecurity, health inequalities, frailty and under nutrition.^27 32^ Secondly, further prompts have been added to address the importance of physical activity and sarcopenia.

The challenges of implementing nutritional care should not equate to overlooking the nutritional health of older adults in the community and we believe a biopsychosocial framework is essential, to acknowledge the complexity of undernutrition with the inclusion of psychosocial factors such as low mood, isolation and food insecurity, not typically addressed despite evidence of significant effects on malnutrition risk.^27^ We hypothesise that recognising difficulties, and identifying interventions that relate to the cause of the problem are key to improving nutrition outcomes and quality of life (see figure 2).

We respond to these challenges by focusing on a ‘what matters to you’ approach^28^ and using a tool that recognises nutrition as essential to life and ageing well.

### Proposed practice pattern

The ‘Nutrition Checklist’ was codeveloped with patients, carers, volunteers and HCPs and the Patients Association and underwent various iterations, following testing and modifications with user feedback.^29–31^ It screens for undernutrition and supports identification of the cause(s) enabling conversation and signposting to appropriate management strategies. The Nutrition Checklist requires no specialist training but has been validated against the MUST and found to have adequate sensitivity and specificity.^30^ It is useful in initiating meaningful conversations with older adults about early (<5%) unintentional weight loss and potential actions. The proposed practice places greater emphasis on psychosocial factors such as the ability to afford, shop and prepare food, rather than acute illness/no nutritional intake >5 days.^32^ By involving patients, and using an interdisciplinary approach, we recognise the importance of awareness and self-care for an ageing population. Additionally, this practice acknowledges the lack of confidence in some HCPs,^16–18 33^ the limited workforce and capacity of GPs and dietetics (in community services and primary care network roles) and the changing primary care workforce.

Using the screening questions (see figure 2 adapted process), any patient, family or primary care HCP can identify potential undernutrition prevention opportunities. The screening tool can be integrated into routine care and existing pathways such as comprehensive geriatric assessment and frailty pathways, and built into local electronic patient records. The checklist focuses on the reasons for risk of malnutrition and guides towards basic advice and signposting to services that can provide support. Ensuring guidance adapted to local provision is essential, and mapping of local services should be reviewed regularly with a designated individual leading at primary care network level. Monitoring and reviewing are essential.

The proposed practice pattern complements local policies, while improving awareness of malnutrition and enhancing the toolkit for subsequent discussions to support management with a focus on aetiology. Similarly, encouraging interdisciplinary use of the checklist is not intended to replace dietetic input. Dietitians can support improved outcomes, using a variety of interventions such as training or support to individual patients and groups.^34^

### Evidence to support practice pattern

We do not anticipate any harms or risks arising from a proactive, patient-centred and aetiology-based approach.

The codevelopment of the checklist with patients >65 years avoids the difficulties of more formal, medicalised screening an identification of malnutrition risk with a lack of shared understanding of what this means^13^ and barriers and facilitators.^35^ The use in frail adults <65 years requires further evaluation, and further refinement of the checklist is recommended to reflect our developing knowledge of holistic, multifaceted interventions and their impact.

The economics of this practice pattern have not been evaluated. It is anticipated that earlier identification is cost-effective. Additionally, aetiology-based intervention may avoid overemphasis on ONS prescribing.^25^ Research bias also impacts evaluation of cost-effectiveness of ONS^25 26 36^ in comparison to non-commercial interventions. Unintentional weight loss is associated with more hospital admissions, visits to GPs and prescriptions therefore this approach might result in reductions in these healthcare outcomes with improvements in patient-related outcomes through:

1. Increase in the frequency and confidence in starting conversations about food and eating behaviours by patients, families and all HCP in primary care.
2. Better use of consultation time on identifying, managing, coaching and addressing undernutrition in primary care.
3. Reductions in unintentional weight loss, frailty and clinical sequelae requiring clinical management in both primary and secondary care.
4. Reduced overdependence on inappropriate or ineffective prescribing of ONS.

Additionally, it supports a proactive approach in clinical nutrition and dietetic practices, away from a traditional commissioning model of reactive treatments, and establishes models with a focus on preventative and earlier interventions, alongside population health management of undernutrition in older adults.

### Summary and perspective section

Existing biomedical approaches to malnutrition are simplistic, do not address the complexity or far-reaching consequences of malnutrition on the health and well-being of older adults adequately. A biopsychosocial model, in the form of a practice pattern, offers a proactive, patient-centred approach for improved outcomes based on relevance to patients, carers and HCPs, and increased utilisation by multidisciplinary teams. Based on this model, further service evaluation of existing practices can provide a ‘Gap Map’ of areas that can be strengthened in practice.

## Data Availability

Data sharing not applicable as no datasets generated and/or analysed for this study. No data are available.
